# A case of colonoscopy-induced Wunderlich's syndrome in a hemodialysis patient: coincidence or association?

**DOI:** 10.1590/2175-8239-JBN-2021-0076

**Published:** 2021-09-13

**Authors:** Antoine Jean Zgheib, Elias Gerges Mansour, Joe Nohra Nohra

**Affiliations:** 1 Maritime Hospital, Department of Nephrology, Jubail, Lebanon.; 2 Notre Dame Maritime Hospital, Department of Urology, Byblos, Lebanon.; 3 Saint George Hospital University Medical Center, Beirut, Lebanon.

**Keywords:** Renal Hemorrhage, Renal Dialysis, Kidney Diseases, Cystic, Colonoscopy, Hemorragia Renal, Hemodiálise, Doença Renal Cística Adquirida, Colonoscopia

## Abstract

Wunderlich syndrome, or spontaneous renal hemorrhage (SRH), is a rare condition encountered in patients undergoing chronic hemodialysis (HD) usually attributed to acquired cystic kidney disease (ACKD) among other causes. In the literature, colonoscopy is associated with splenic injuries, and renal hemorrhage has not been previously described. Management can range from conservative treatment to angiographic embolization or exploration and nephrectomy. Here we report an unusual case of a 54-year-old woman HD patient who presented with SRH within a few days of colonoscopy. The reason of SRH was rupture of an ACKD cyst. We assumed that colonoscopy was a provoking factor and elaborated hypotheses for its etiopathogenesis. The patient underwent successful left nephrectomy. The importance of this case lies in the fact that colonoscopy is not always an innocent procedure in HD patients, and could be complicated by renal cyst hemorrhage.

## Resumo

A síndrome de Wunderlich, ou hematoma perirrenal espontâneo (HPE), é uma condição rara encontrada em pacientes submetidos à hemodiálise crônica (HD) geralmente atribuída à doença renal cística adquirida (DRCA), entre outras causas. Na literatura, a colonoscopia está associada a lesões esplênicas, e o hematoma renal não foi descrito anteriormente. O manejo pode variar de tratamento conservador a embolização angiográfica ou exploração e nefrectomia. Aqui relatamos um caso incomum de uma paciente em HD de 54 anos de idade que se apresentou com HPE dentro de poucos dias após a colonoscopia. O motivo do HPE foi a ruptura de um cisto de DRCA. Consideramos que a colonoscopia foi um fator provocador e elaboramos hipóteses para sua etiopatogenia. A paciente foi submetida a uma nefrectomia esquerda bem-sucedida. A importância deste caso reside no fato de que a colonoscopia nem sempre é um procedimento inocente em pacientes em HD, e pode ser complicada por hemorragia do cisto renal.

## Introduction

Wunderlich syndrome, also known as spontaneous renal hemorrhage (SRH), is a rare and potentially fatal condition in patients undergoing hemodialysis (HD) that is attributed to acquired cystic kidney disease (ACKD). Other causes include angiomyolipoma and renal cell carcinoma (RCC), while vascular entities such as polyarteritis nodosa and Wegener polyangiitis are rare^1^. Most published cases following colonoscopy discuss splenic rupture as unusual complication, and may present in 1-21/100,000 of cases^2^. To the best of our knowledge, SRH has never been described before as a complication of renal cyst rupture in ACKD after colonoscopy. We describe a HD patient with ACKD who developed SRH after colonoscopy along with a discussion of the contributing factors and highlight that HD patients should be under close surveillance after gastrointestinal endoscopy. We report this case because of its rarity and significance with respect to being a complication in a dialysis patient.

## Case Presentation

**Figure 1:**
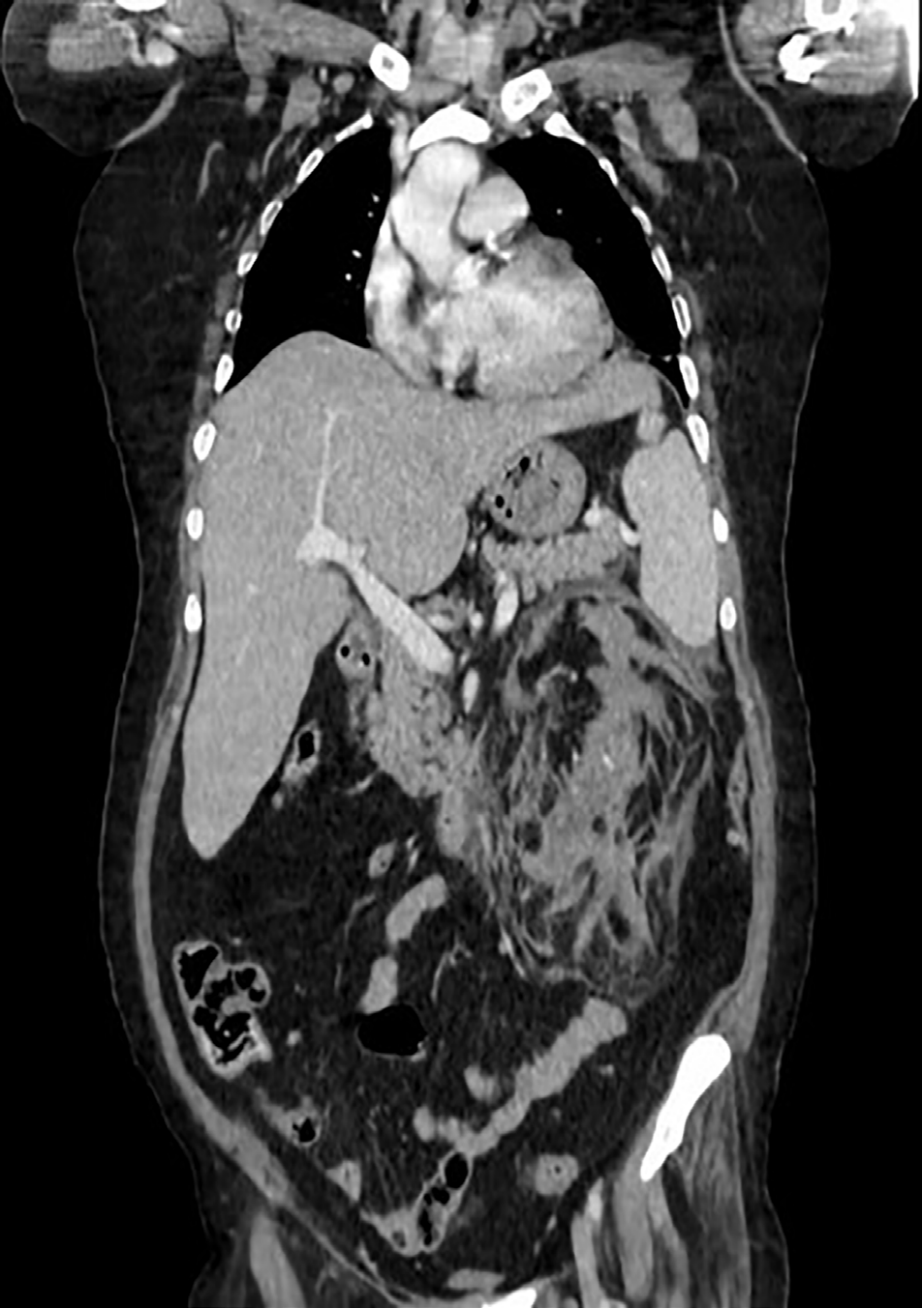
Coronal CT scan section showing the left perirenal intraparenchymal hematomas with a subcapsular collection of up to 1.8-cm thick with hematic collection throughout the renal compartment reaching a diameter of more than 9×4 cm.

**Figure 2:**
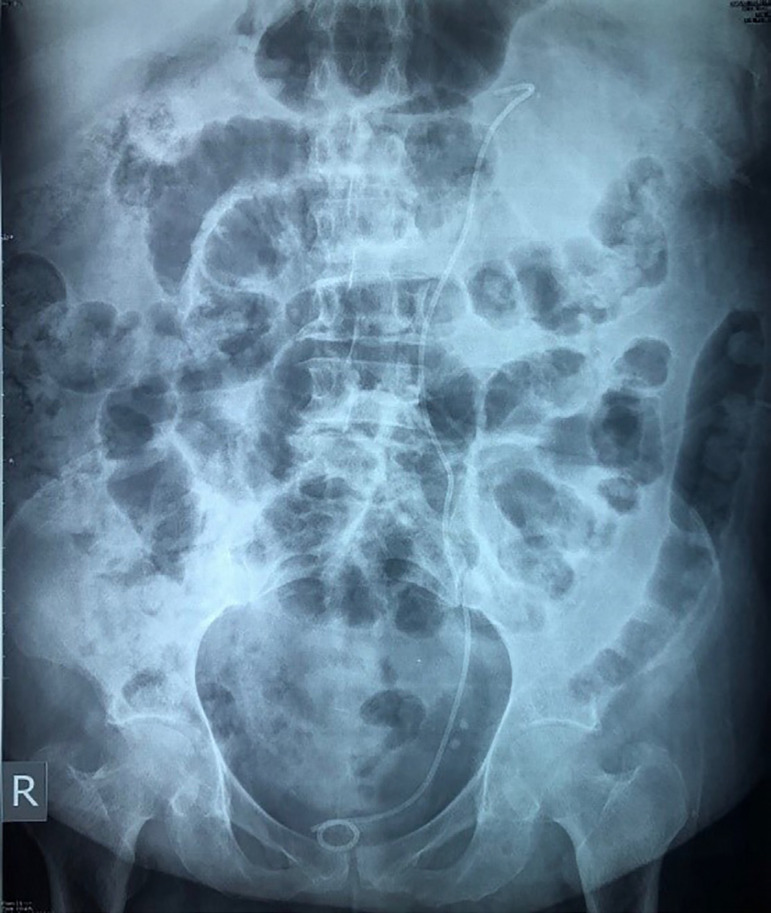
X-ray KUB showing the left JJ in place, as well as the diffuse colonic distention corresponding to ileus post colonoscopy.

A 54-year-old Lebanese woman, on maintenance hemodialysis (HD) trice weekly for focal segmental glomerulosclerosis (FSGS) was admitted to the emergency department, after she had undergone her scheduled hemodialysis the day before, for sudden onset of severe left flank pain, nausea, and vomiting. The pain had started suddenly about 12 hours prior to presentation and worsened rapidly. On a side note, a severe secondary hyperparathyroidism with cutaneous calciphylaxis was treated by cinacalcet for 3 years without response required parathyroidectomy (four glands hyperplasia) 8 months before her current admission. Five days prior to presentation, she was admitted to the hospital because of severe anemia and rectorrhagia. Her renal ultrasound evaluation showed bilateral small kidneys with thinned parenchyma containing cysts of up to 3 cm and vascular calcifications without hydronephrosis. Four days prior to presentation, colonoscopy through terminal ileum revealed a 7 mm polyp in the left descending colon which was removed by mucosectomy. Microscopy returned as sessile tubular adenoma with low-grade dysplasia. The patient denied fever or a history of trauma. There was no previous history of antiplatelet use. In the emergency department, her blood pressure and pulse rate were 112/65 mmHg and 96 bpm, her temperature was 36.8^°^C and she had mildly distended abdomen with severe left costovertebral angle tenderness. Laboratory findings showed hemoglobin of 9.0 gm/dL while hematocrit was 29.7%, total leucocyte count 12.2 × 1000/microliter, creatinine of 6.06 mg/dL, and C-reactive protein level of 6 mg/L. In addition, PT was 83% and INR was 1.13. On the day of presentation (i.e, 4 days after colonoscopy), a multiphasic computed tomography (CT) scan of the thorax, abdomen, and pelvis was performed with and without intravenous contrast. The scan revealed a large cyst and huge distorting left perirenal intraparenchymal hematomas with a subcapsular collection of up to 1.8 cm thick with hematic collection throughout the renal compartment reaching a diameter of more than 9×4 cm, left hydronephrosis, and edema surrounding fat throughout the flank and calcifications in the hilum (Figure 1). For decompression of the renal pelvicalyceal system and drainage, we chose to treat her with retrograde left double-J ureteral stent insertion which identified a clot in the left mid-ureter, and urine culture and cytology were taken and returned negative. Plain film of abdomen showed the double J in place (Figure 2). The patient remained hemodynamically stable, but had a further drop in hemoglobin (6.1 g/dL) the same evening, which required a transfusion of one unit of leuco-depleted red cell concentrate for stabilization. A decision was made for surgical exploration with presumptive diagnosis of subacute left perirenal hematoma and for exclusion of underlying malignancy. She underwent a HD session without heparin. On day 3, she underwent open radical left nephrectomy with conservation of the adrenal gland. She received 2 more units of packed leuco-depleted red cell concentrate during the intervention. The renal parenchyma was almost entirely replaced by a hematoma measuring 9×9×7 cm and extending into the peri-renal fat. A small papillary mass was sent separately for analysis. The detailed histopathological evaluation revealed a renal parenchyma with lesions of chronic nephritis mutilated by large hemorrhagic areas which extended to the peri-renal fat. In addition, there was a neoplastic proliferation measuring 2 mm, with papillary architecture covered by little atypical cubic cells and foamy macrophages in the axis compatible with papillary adenoma. On the next operative day the patient underwent HD and received a pack of leuco-depleted red blood cells. The post-operative period was uneventful. Hemoglobin level remained around 10 g/dL. She was discharged on the seventh day without any bleeding sign or symptoms. She remains well and asymptomatic and is currently on regular maintenance HD.

## Discussion

Since it was first described in 1856, spontaneous renal hemorrhage (SRH) has been reported infrequently in the literature, where dialysis patients are often excluded. Classic symptoms, such as acute flank pain, flank mass, and signs of internal bleeding, known as Lenk's triad, have limited sensitivity and specificity for detecting SRH^3^. Nowadays, computed tomography (CT) is the method of choice for the diagnosis. Most frequent etiologies are renal cysts, benign and malignant renal tumors, vascular lesions, and antiplatelet or anticoagulant therapy^3^. While benign and malignant tumors can be causes of renal bleeding, a tiny 2-mm adenoma cannot solely explain the severe bleeding in our case especially that the histopathological exam did not reveals signs of intra-tumoral bleed.

ACKD is a well-known late stage complication of end-stage renal disease (ESRD). It can develop in up to 74% of patients with end-stage renal failure undergoing chronic HD lasting more than four years^3^ .The prevalence of ACKD is directly related to the duration of dialysis and cysts tend to grow with time. Numerous complications have been described in relation to this entity including lithiasis, infection, urinary tract obstruction, malignant degeneration, and intracystic bleeding that can cause a spontaneous retroperitoneal hemorrhage (RH). Hemorrhagic renal cysts are the most frequent complication of patients with ACKD^4^. Bleeding is usually confined within the cyst but occasionally extends into the kidney collecting system leading to hematuria or into the perinephric space leading to perirenal hematoma or RH. Perinephric hematomas have been reported in up to 13% of patients with ACKD^3^. Among dialysis patients, ACKD has been recognized to be the most frequent cause. Approximately 50% of patients with ACKD develop hemorrhagic renal cysts.^4^ Malek-Marín et al. reported a single center incidence of SRH in hemodialysis patients of 0.86 cases/100 patients.^5^ When SRH was of renal origin in dialysis patients, the most common cause was cyst rupture in patients with ACKD^5^. The usual definition of ACKD requires three or more cysts per kidney in a patient on dialysis who does not have a hereditary cause of cystic disease. Within the first 3 years of dialysis, approximately 10-20% of patients develop ACKD. By 5 years, 40-60% of patients have ACKD and by 10 years more than 90% of patients exhibit ACKD^6^. Our patient had risk factors for development of ACKD, including long duration of renal replacement therapy. Moreover, our patient did not have any cysts in the kidney at the start of the dialysis. Nephrectomy material also histologically confirmed the diagnosis of ACKD. It is well documented that pre-existing renal disease can predispose a kidney to injury. Another notable observation in SRH secondary to cyst rupture is that there is a remarkable difference in frequencies between SRH due to ACKD and autosomal dominant polycystic kidney disease (ADPKD). Most of the described patients in the literature had underlying ACKD rather than ADPKD. Some putative factors have been put forward to explain this disparity. The more medullary location of cysts in ADPKD compared with cortically residing cysts of ACKD and differences in the speed of cyst growth might account for differences in observed prevalence of SRH^5^. Among hemodialysis patients, perirenal or retroperitoneal bleeding due to acquired cyst rupture tends to occur after the dialysis session, possibly as a consequence of heparinization^6^.

Although the mechanism of SRH in the present case remains elusive, several factors might have contributed to the bleeding. First, the histories of hyperparathyroidism, uremia, chronic HD, and atrophied kidney with cystic change of ACKD were risks for hemorrhage. Second, the patient received her heparin dose the day before the colonoscopy. Third, the presence of calciphylaxis, also known as calcific uremic arteriolopathy, should also be considered. It has been reported in 1 to 4.5% of patients in dialysis with vascular calcifications with calcium oxalate crystals tissues depositions^7^. Our patient had diffused small artery calcification and calcium oxalate crystals in her left nephrectomy specimen and her skin lesions of calciphylaxis healed progressively after parathyroidectomy. Fourth, it would be difficult to establish that maneuvers performed during colonoscopy and endoscopic mucosal resection (EMR) were the cause of SRH due to the anatomical proximity the left kidney which is posterior to the descending colon. We believe that elevated intraluminal pressure as well as increased intraperitoneal pressure from gas insufflation during and external pressure on the left hypochondrium exacerbated by ileus from deep sedation, since patients cannot report pain associated with stretching, also may contribute to increased perinephric pressure. Finally, it should be pointed out that renal cysts of ACKD are associated with fragile vessels focally calcified stretched across their distended walls, as in our patient, with thickened hypertrophic intima and fibrosis. When intracystic pressure rises, these vessels may leak blood into the cyst, causing it to expand rapidly, resulting in severe pain. If bleeding continues, then the cyst may rupture into the collecting system or, alternatively, it may rupture into the subcapsular compartment and eventually pass through the renal capsule to fill the retroperitoneal space. 

Despite the fact that this case sheds light on a serious complication that may be related to colonoscopy, the correlation between the conditions needs further investigations and the pathophysiology remains a hypothesis that needs validation especially with the incidental finding of the renal adenoma that could contribute to the bleed. Nevertheless, most of the clinically overt SRH cases in dialysis patients are secondary to rupture of acquired cysts, especially after 5 years of renal replacement therapy, hence, patients should be screened for the presence of ACKD; this surveillance should be continued because of risk of cyst enlargement and malignancy. Because of the high probability of small clinical non-apparent underlying malignancy in HD patients who have ACKD with SRH, nephrectomy was necessary in our anuric patient.

## Conclusion

SRH is a rare but important and potentially fatal complication. Its frequency is most probably underestimated in daily practice. We described a HD patient with ACKD who developed SRH after colonoscopy and we believe that this procedure has not been reported before as an underlying precipitating etiology. The reason of SRH was rupture of ACKD cysts. HD patients should be under close surveillance related to ACKD development and associated bleeding risks after colonoscopy. Finally, reporting of similar cases in medical literature seems necessary to shed more light on this obscure entity and increase the awareness among clinicians.
